# Mir-573 regulates cell proliferation and apoptosis by targeting Bax in human degenerative disc cells following hyperbaric oxygen treatment

**DOI:** 10.1186/s13018-020-02114-6

**Published:** 2021-01-07

**Authors:** Song-Shu Lin, Chi-Chien Niu, Li-Jen Yuan, Tsung-Ting Tsai, Po-Liang Lai, Kowit-Yu Chong, Kuo-Chen Wei, Chiung-Yin Huang, Meng-Ling Lu, Chuen-Yung Yang, Steve W. N. Ueng

**Affiliations:** 1 Department of Orthopaedic Surgery, Chang Gung Memorial Hospital, No 5, Fu-Hsing Street, Linkou, Taoyuan, 333 Taiwan; 2 Department of Nursing, Chang Gung University of Science and Technology, Taoyuan, Taiwan; 3 Hyperbaric Oxygen Medical Research Lab, Bone and Joint Research Center, Chang Gung Memorial Hospital, Taoyuan, Taiwan; 4 College of Medicine, Chang Gung University, Taoyuan, Taiwan; 5 Department of Orthopaedic Surgery, E-Da Hospital/I-Shou University, Kaohsiung, Taiwan; 6 Department of Medical Biotechnology and Laboratory Science, College of Medicine, Chang Gung University, Taoyuan, 333 Taiwan; 7 Department of Neurosugery, New Taipei Municipal Tu Cheng Hospital, Chang Gung Memorial Hospital and Chang Gung University, Taoyuan, Taiwan; 8 Department of Orthopaedic Surgery, Chang Gung Memorial Hospital, Kaohsiung, Taiwan

**Keywords:** MicroRNA-573, Bax, Caspase, HBO, NP

## Abstract

**Background:**

MicroRNA (miRNA) plays a vital role in the intervertebral disc (IVD) degeneration. The expression level of miR-573 was downregulated whereas Bax was upregulated notably in human degenerative nucleus pulposus cells. In this study, we aimed to investigate the role of miR-573 in human degenerative nucleus pulposus (NP) cells following hyperbaric oxygen (HBO) treatment.

**Methods:**

NP cells were separated from human degenerated IVD tissues. The control cells were maintained in 5% CO_2_/95% air and the hyperoxic cells were exposed to 100% O_2_ at 2.5 atmospheres absolute. MiRNA expression profiling was performed via microarray and confirmed by real-time PCR, and miRNA target genes were identified using bioinformatics and luciferase reporter assays. The mRNA and protein levels of Bax were measured. The proliferation of NPCs was detected using MTT assay. The protein expression levels of Bax, cleaved caspase 9, cleaved caspase 3, pro-caspase 9, and pro-caspase 3 were examined.

**Results:**

Bioinformatics analysis indicated that the 3′ untranslated region (UTR) of the Bax mRNA contained the “seed-matched-sequence” for hsa-miR-573, which was validated via reporter assays. MiR-573 was induced by HBO and simultaneous suppression of Bax was observed in NP cells. Knockdown of miR-573 resulted in upregulation of Bax expression in HBO-treated cells. In addition, overexpression of miR-573 by HBO increased cell proliferation and coupled with inhibition of cell apoptosis. The cleavage of pro-caspase 9 and pro-caspase 3 was suppressed while the levels of cleaved caspase 9 and caspase 3 were decreased in HBO-treated cells. Transfection with anti-miR-573 partly suppressed the effects of HBO.

**Conclusion:**

Mir-573 regulates cell proliferation and apoptosis by targeting Bax in human degenerative NP cells following HBO treatment.

## Background

Intervertebral disc degeneration (IDD) is considered to be the pathological basis of degenerative spinal diseases, leading to intervertebral disc herniation, spinal canal stenosis, and lower back pain [1]. Apoptosis is a key component responsible for the decrease in the cell number of nucleus pulposus cells during degeneration [2, 3]. There were two apoptotic factors Bax (pro-apoptotic protein) and Bcl-2 (anti-apoptotic protein); the former binds to the mitochondrial membrane and induces the release of cytochrome c [4] and the latter prevents the formation of Bax homodimers [5], which inhibits the cytosolic accumulation of cytochrome c and activation of caspase 3 [6]. Bcl-2 prevents or delays apoptotic induction by a large variety of stimuli in many cell types [7]. Overexpression of bcl-2 in intervertebral disc (IVD) cells reduced the mRNA expression level of caspase 3 and prevented in vitro apoptotic cell death [8].

MicroRNAs (miRNAs) are endogenous non-coding small RNAs consisting of 20–25 nucleotides that serve to mediate gene regulatory events by pairing with the 3′ untranslated region (3′ UTR) of their target messenger RNAs (mRNAs) and, thus, modulating their expression [9]. Various miRNAs are dysregulated in IDD and functionally implicated in its pathogenesis. MiR-21 promotes human NP cells proliferation by affecting PTEN/AKT signaling [10]. MiR-494 induced cell apoptosis via directly combining with SOX9 in human degenerative NP cells [11]. Therefore, miRNAs might play an important role in the development and progression of IDD through regulating NP cells proliferation and apoptosis.

Human IVD is the largest avascular tissue in the body [12]. During degeneration, structural changes with vascular depletion, endplate calcification, and disc size increase make oxygen diffusion harder and the oxygen concentration in the IVD becomes even lower [13]. Hyperbaric oxygen (HBO) treatment can serve to improve hypoxic conditions by increasing tissue and/or microvascular O_2_ levels [14]. The expression level of miR-573 was downregulated whereas Bax was upregulated notably in degenerative NP cells [15]. However, the role of miR-573 in degenerative NP cells following HBO treatment has not been fully elucidated.

In this study, we demonstrated that HBO treatment increased miR-573 expression in degenerated NP cells, as assessed via microarray analysis and confirmed by real-time PCR. We used bioinformatics to identify putative target sequences for miR-573 in the human Bax mRNA and confirmed these by way of luciferase reporter assays. Subsequently, we examined the cell proliferation, Bax protein, cleaved-caspase 9 protein, pro-caspase 9 protein, cleaved-caspase 3 protein, and pro-caspase 3 protein expression after HBO treatment. Our findings provide a new therapeutic target for the HBO treatment of IDD.

## Materials and methods

The experimental protocol was approved by the Human Subjects Institutional Review Board at Chang Gung Memorial Hospital, Taiwan.

Preparation of human primary intervertebral disc tissue cell cultures. Fresh abnormal disk tissue was harvested from the degenerated lumbar IVD of 28 patients (male = 10, female = 18, age = 63.1 ± 12.7) who receive total discectomy and posterior lumbar interbody fusion (PLIF). During the operation, the abnormal disc was explored by partial laminotomy and total facetectomy at each side. After proper protection of the existing and traversing nerve roots, the disc tissue, both annulus and nucleus, was removed massively by disc rongeur through a discotomy hole and along the path of the cage in each side until the endplates were well exposed. NP cells were separated from the nucleus tissues by performing sequential enzymatic digestion, first with 0.4% pronase (Sigma) for 1 h and subsequently with 0.025% collagenase P (Boehringe) and 0.004% DNase II (Sigma) at 37 °C overnight. After digestion, the cells were washed with DMEM/F-12 and seeded in three fresh flasks at a density of 5000 cells/cm^2^ and incubated in a humidified atmosphere of 5% CO_2_ and 95% air until the cells attained confluence. Cells were used at passage 2 for each experiment.

### Cells exposure to intermittent HBO

Approximately 2 × 10^5^ cells were plated on a 100-mm cell culture dish containing 10 mL DMEM/F-12 supplemented with 10% FBS. The cultures were maintained at 37 °C in a humidified atmosphere of 5% CO2 and 95% air. The cells were either maintained in 5% CO_2_/95% air throughout the experiment as a control or in HBO-treated protocol containing three times of 100% O2 at 1.5 atm (atmosphere) for 25 min each, two times of air break (5% CO2/95% air) at 1.5 atm for 5 min each in a hyperbaric chamber (Sigma II; Perry Baromedical, USA). The total duration for HBO-treated protocol is about 120 min. HBO treatment administered a total of 120 min every 48 h.

### MicroRNA profiling

Total RNA was extracted from cells using mirVana miRNA isolation kit (Ambion, Austin). MiRNA expression profiling was accomplished using TaqMan Human MicroRNA Array B Cards containing 384 mature human microRNAs (Applied Biosystems, USA) and an ABI 7900 real-time PCR System according to the manufacturer’s protocol. MiRNA expression profiling was performed on eight samples from four patients (with or without HBO treatment). Briefly, 3 μL of total RNA from each sample was reverse-transcribed using the Taq-Man miRNA Reverse Transcription Kit (Applied Biosystems, USA) and the stem-loop Megaplex Primer Pool Sets. A total of 7.5 μL of reaction mixture was immediately incubated under the following conditions: 40 cycles at 16 °C for 2 min, 42 °C for 1 min, 50 °C for 1 s, and 85 °C for 5 min. Then, 2.5 μL of the resultant Megaplex RT products were mixed with 2.5 μL of Megaplex PreAmp Primers and 12.5 μL of TaqMan PreAmp Master Mix. A total of 25 μL of the reaction mixture was incubated using the following program: 95 °C for 10 min, 55 °C for 2 min, and 72 °C for 2 min followed by 12 cycles at 95 °C for 15 s, 60 °C for 4 min, and 99.9 °C for 10 min. The pre-amplified cDNA was diluted with 0.1× TE (10 mM Tris, pH 8.0, 1 mM EDTA) to 100 μL and used for PCR. The relative miRNA expression levels were calculated by the 2^-ΔΔCt^ method as follows: ΔCt (test) = Ct (miRNA of interest, test) − Ct (internal reference, test), ΔCt (calibrator) = Ct (miRNA of interest, calibrator) − Ct (internal reference, calibrator), ΔΔCt = ΔCt (test) − ΔCt (calibrator). The inter-individual variability of the efficiency of our procedures was controlled by spiking of U6 snRNA. The hierarchical cluster analysis of differentially expressed miRNAs was performed using CLUSTER 3.0 [16], and the hierarchical clustering heat map was visualized by Tree View [17].

### Real-time PCR

TaqMan miRNA assays (ABI PRISM; Applied Biosystems, USA) were used to detect the expression levels of the mature miR-573. For the reverse transcription (RT) reactions, 10 ng of total RNA was mixed with the RT primer. RT reactions were performed at 16 °C for 30 min, 42 °C for 30 min, 85 °C for 5 min, and then maintained at 4 °C. Following the RT reactions, 1.5 μL of cDNA was used for a polymerase chain reaction (PCR) using 2 μl of TaqMan primers. The PCR was conducted at 95 °C for 10 min followed by 40 cycles of 95 °C for 15 s and 60 °C for 60 s in an ABI 7900 real-time PCR system (Applied Biosystems, USA). The fold change in the miRNA expression in each sample relative to the average expression in the control was calculated based on the threshold cycle (CT) value using the 2^-ΔΔCt^ method.

### MiRNA target prediction and dual-luciferase reporter assays

Target Scan 7.2 (http://www.targetscan.org) online software was used to analyze the putative target genes of miR-573. The 3′ UTR of Bax containing the miR-573 binding site was cloned into pmirGLO dual-luciferase miRNA reporter vectors (Promega, USA). A mutated 3′ UTR of Bax was introduced into the potential miR-573 binding site. The reporter vectors containing the wild-type or mutant Bax 3′ UTR were transfected into NP cells using Lipofectamine 3000 (Invitrogen, USA). After incubation with or without HBO, transfected cells were lysed. Firefly and Renilla luciferase activities were detected using the dual-luciferase assay system (Promega, USA) in accordance with the manufacturer’s instructions.

### Transfection of NP cells with anti-miRNAs and analysis after HBO treatment

NP cells were seeded into 24-well plates at a density of 2 × 10^4^ cells/cm^2^ in culture medium without antibiotics and divided into control, HBO, and HBO + anti-miR-573 groups. The next day (day 1), cells were transfected with anti-miR-573 (100 nM; Ambion, USA) using RNAiMAX (Invitrogen, USA) and cultured in an incubator at 37 °C with 5% CO_2_. After 8 h of transfection, the culture medium was changed to DMEM/F-12 with 5% FBS, and the cells were exposed to HBO treatment. Cell proliferation was measured using the 3-(4,5-dimethyl-2-thiazolyl)-2,5-diphenyl-2H tetrazolium bromide (MTT) assay. Forty-eight hours after HBO treatments, cells were washed once in serum-free DMEM/F-12 medium, then 900 μL/well medium and 100 μL/well MTT (5 mg/mL in PBS; Sigma) were added for 4 h at 37 °C. The dye solution was removed and cells washed once with PBS before adding 1000 μL/well dimethyl sulfoxide (DMSO) and mixing in an orbital shaker for 30 to 60 min at room temperature in the dark. Finally, 150 μL samples were removed from each well and put into 96-well plates, and absorbance was read at 570 nm against a blank of DMSO using a microplate reader (Dynex MRX; Dynex Technologies Ltd., UK). Data expressed the (absorbance value) ratio of HBO and HBO + anti-miR-573 to the control group.

NP cells were seeded into six-well plates at a density of 2 × 10^5^ cells/well in culture medium without antibiotics. The next day (day 1), cells were transfected with anti-miR-573 (100 nM) using RNAiMAX (Invitrogen) and cultured in an incubator at 37 °C with 5% CO_2_. After 8 h of transfection, the culture medium was changed to DMEM/F-12 with 5% FBS, and the cells were exposed to HBO treatment. On days 3 and 5, the cells were re-transfected once and exposed to HBO. At 12 h after the third HBO treatment, cellular RNA was isolated using an RNeasy mini kit (Qiagen, USA) and reverse-transcribed into cDNA with the ImProm-II reverse transcription system (Promega, USA) for real-time PCR detection of Bax transcripts. At 24 h after the third HBO treatment, cells were washed with PBS and extracted using M-PER mammalian protein extraction reagent (Thermo Fisher Scientific, USA) for immunoblotting experiments. The proteins were separated via SDS-PAGE and transferred onto nitrocellulose membranes using a protein transfer unit (Bio-Rad, USA). After blocking with 10% nonfat milk, the membranes were incubated overnight at 4 °C with a 1000-fold dilution of mouse antibodies against Bax (Abcam, UK) or β-actin (Abcam, UK) or a 1000-fold dilution of rabbit antibodies against procaspase-9, cleaved-caspase-9, procaspase-3, or cleaved-caspase-3 (Cell Signaling Technology, MA). After washing, the membranes were further incubated for 2 h with a 10,000-fold dilution of goat anti-mouse IgG conjugated to horseradish peroxidase (CalBiochem, USA) or goat anti-rabbit IgG conjugated to horseradish peroxidase (CalBiochem, USA). The membranes were then washed and rinsed with ECL detection reagents (Amersham Pharmacia Biotech, UK). The band images were photographed using Hyperfilm (Amersham Pharmacia Biotech, UK). The intensity of the staining for Bax, procaspase-9, cleaved-caspase-9, procaspase-3, cleaved-caspase-3, and β-actin was quantified using an image analysis system (Image-Pro plus 5.0; Media Cybernetics, USA).

### Statistical analysis

Data are expressed as mean ± standard deviation (SD). The *p* value for the Student’s *t* test was calculated, and a *p* value of < 0.05 was considered statistically significant.

## Results

### Heat maps of miRNA expression in degenerated NP cells after HBO treatment

**Fig. 1:**
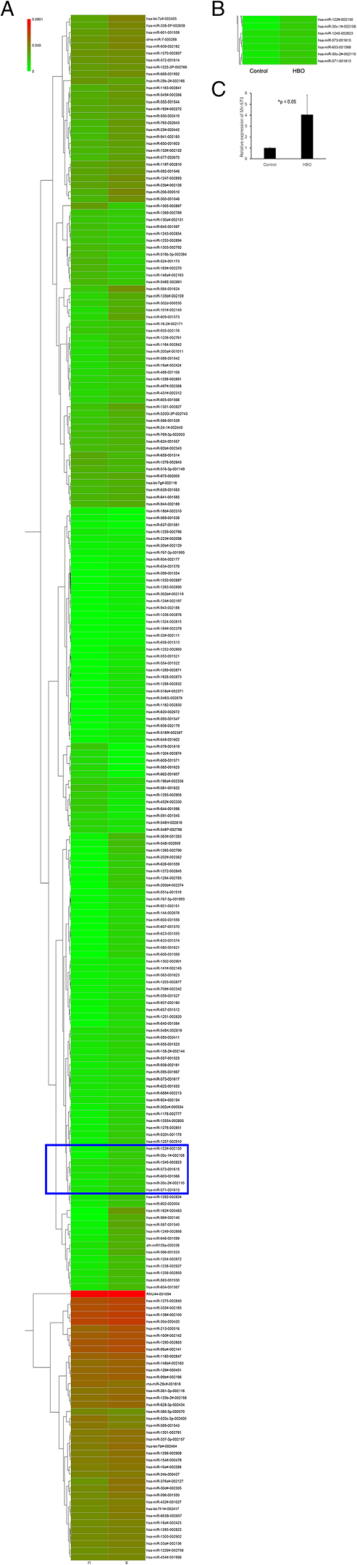
Hierarchical cluster of miRNAs. HBO modulates the expression of miRNAs in degenerated NP cells. **a** A heat map of the miRNAs with significantly changed expression levels in the HBO-treated group compared with the control group. Average expression value is shown for each miRNA in each class. The red and green colors indicate that the ΔCt value is below (relatively high expression) and above (relatively low expression levels) the median of all ΔCt values. **b** MiR-573 was chosen for further investigation. **c** HBO treatment increased miR-573 expression in NP cells (*p < 0.05)

To identify the miRNAs involved in the molecular regulation of NP cells after HBO treatment, the miRNA expression profile of NP cells was performed using a TaqMan Human MiRNA Array B Card. As shown in Fig. 1a, there were 185 miRNAs upregulated and 37 downregulated by at least 1.5-fold following HBO treatment. Among these, miR-573 was chosen for further investigation (Fig. 1b, Table 1, *n* = 4) as previous studies revealed that miR-573 expression is lower in degenerative NPCs [15].

**Table 1:** Differentially expressed of miRNA-573 in NPCs following HBO treatment

Detector	Mean_DeltaCt_C	Expression_C	Mean_DeltaCt_H	Expression_H	Delta_Mean_DeltaCt	Fold_Change	Regulation
hsa-miR-573-001615	23.67	7.48E−08	22.07	2.28E−07	− 1.61	3.04	Up

*C* control group, *H* HBO group

### HBO treatment increased miR-573 expression in degenerated NP cells

HBO treatment increased miR-573 expression in NPCs (4.05 ± 1.78-fold, *p < 0.05, *n* = 4; Fig. 1c). These results indicated that miR-573 might play an important role in inhibiting the progression of IDD in NPCs after HBO treatment.

### Seed sequence of miR-573 in the 3′ UTR of the Bax mRNA

**Fig. 2:**
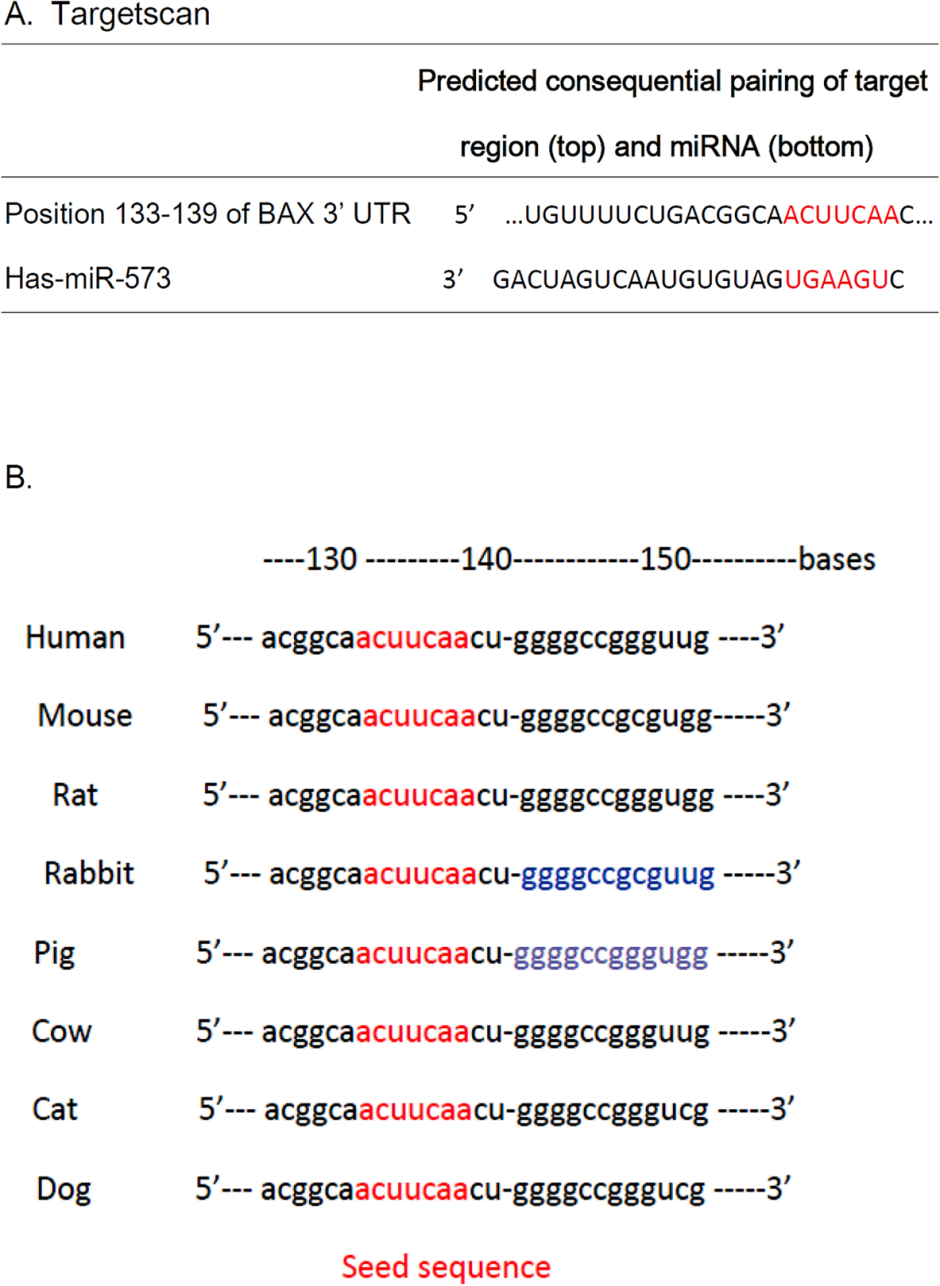
Seed sequence of miR-573 in the 3′ UTR of the Bax mRNA. Target Scan predicted the duplex of miR-573 with the seed sequence in the 3′ UTR of the human Bax mRNA. The sequences in white are the locations of the potential seed-matched sequences for the miRNAs assessed. Cross-species conservation of the miR-573 seed sequence in the 3′ UTR of the human Bax mRNA as identified via the Target Scan algorithm (sequences in red)

To investigate the potential molecular targets of miR-573, we screened for putative target genes of miR-573 using Target Scan 7.2 (http://www.targetscan.org) online software. We found that Bax, an important regulator of apoptosis, was likely a direct target of miR-573, as the 3′ UTR of Bax contained a potential binding element for miR-573 with a 7-nt match to the miR-573 seed region (Fig. 2a). Additionally, cross-species conservation of the miR-573 seed sequence in the 3′ UTR of the Bax mRNA was confirmed by the Target Scan algorithm (Fig. 2b). These findings suggested that hsa-miR-573 might target the Bax mRNA by directly recognizing its seed-matched sequence present in the 3′ UTR.

### Bax is a direct target of miR-573

**Fig. 3:**
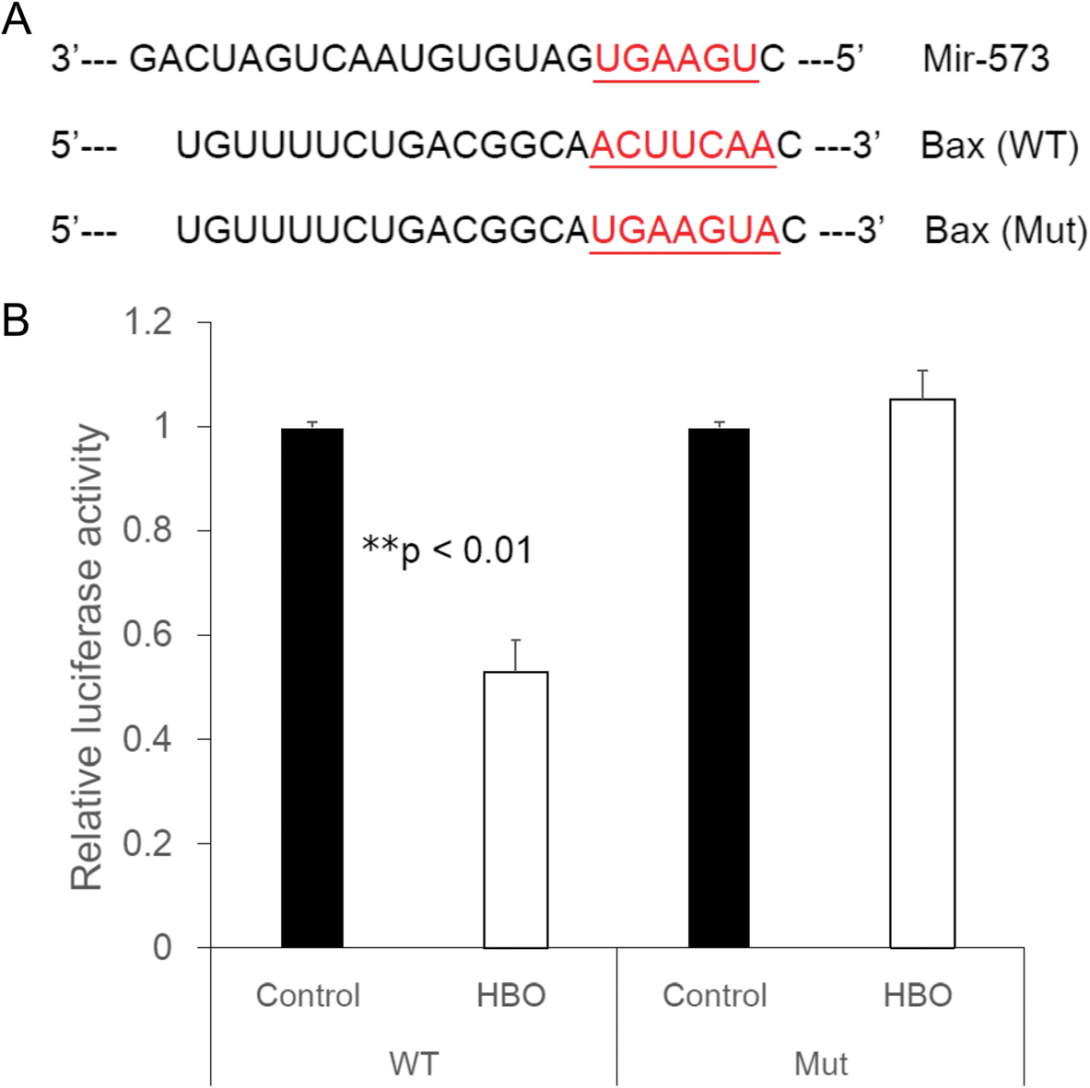
Bax is a direct target of miR-573. Diagram of the binding site between miR-573 and the Bax 3′ UTR. The reporter vectors contain the wild-type (WT) or mutant (Mut) Bax 3′ UTR. Dual-luciferase reporter assay of the Bax 3′ UTR. The reporter vectors containing the WT or Mut Bax 3′ UTR were transfected into NP cells. Luciferase activity was shown to be significantly downregulated after HBO treatment (**p < 0.01; *n* = 4) in the constructs harboring the WT but not in the Mut 3′ UTR (*p* > 0.05, *n* = 4)

To validate the direct targeting of Bax by miR-573, the wild type (WT) or a mutant variant (Mut) of the Bax 3′ UTR containing the target sequence was cloned into a dual-luciferase reporter vector (Fig. 3a). Overexpression of miR-573 after HBO treatment significantly inhibited luciferase activity of the WT Bax 3′ UTR (Fig. 3b, 0.53 ± 0.06-fold, **p < 0.01, *n* = 4), whereas mutation of the miR-573 binding sites abolished this inhibitory effect of miR-573 in the degenerated human NPCs (Fig. 3b, 1.05 ± 0.05-fold, *p* > 0.05, *n* = 4). These observations support the conclusion that Bax is a target gene of miR-573 following HBO treatment.

**Fig. 4:**
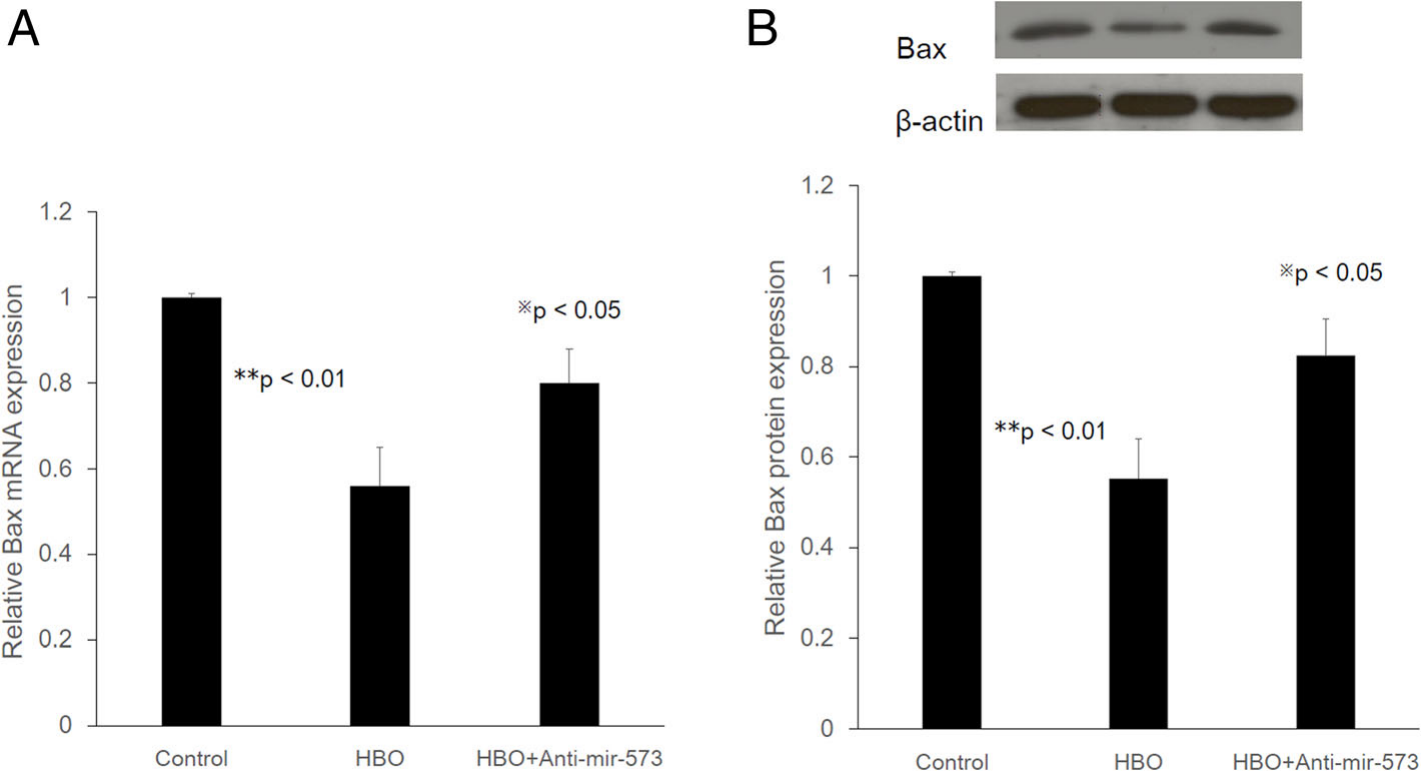
Real-time PCR and Western blot analysis of Bax expression in degenerated NP cells transfected with miR-573 inhibitors following HBO treatment. **a** Bax mRNA expression was down-regulated after HBO treatment (**p < 0.01; *n* = 4). Anti-miR-573 partly reversed the suppressive effects of HBO (^※^*p* < 0.05; *n* = 4). **b** Bax protein expression was significantly down-regulated after HBO treatment (**p < 0.01; *n* = 4). MiR-573 inhibitors partly reversed the suppressive effects of HBO (^※^*p* < 0.05; *n* = 4). Values were normalized against β-actin. WT, wild type; Mut, mutant

We next examined the expression of Bax in NP cells transfected with an anti-miR-573 construct. As shown in Fig. 4a, HBO treatment decreased the mRNA expression of Bax (HBO/control: 0.56 ± 0.09-fold, **p < 0.01, *n* = 4), whereas transfection with anti-miR-573 partly suppressed the effects of HBO treatment (HBO + anti-miR573/control: 0.80 ± 0.08-fold, ^**※**^*p* < 0.05, *n* = 4). Western blot analysis was performed to examine the protein level of Bax (Fig. 4b), and the results indicated that HBO treatment led to a significant decrease in the protein level of Bax (HBO/control: 0.55 ± 0.08-fold, **p < 0.01, *n* = 4), whereas knockdown of miR-573 partly suppressed the effects of HBO treatment (HBO + anti-miR573/Control: 0.84 ± 0.06-fold, ^**※**^*p* < 0.05, *n* = 4). These data indicated that Bax was negatively mediated by miR-573 at the post-transcriptional level in NPCs after HBO treatment, as overexpression of miR-573 after HBO treatment significantly inhibited the mRNA (Fig. 4a) and protein (Fig. 4b) expression of Bax in these cells.

### Overexpression of miR-573 by HBO treatment increases cell proliferation in degenerative NP cells

**Fig. 5:**
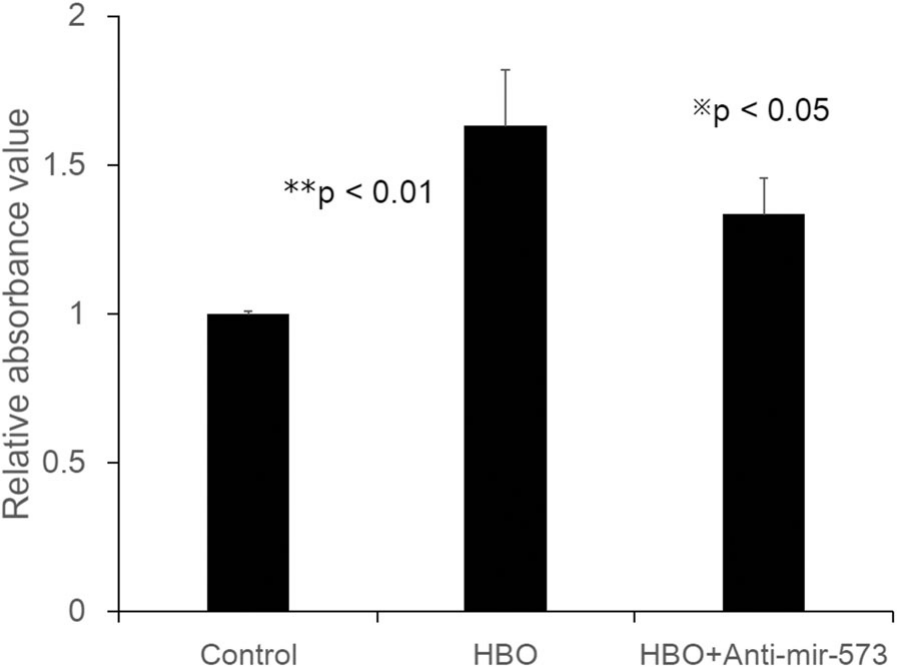
Overexpression of miR-573 by HBO treatment increases cell proliferation in degenerative NP cells. HBO treatment markedly increased NP cells proliferation (**p < 0.01, *n* = 4), whereas transfection with anti-miR-573 partly suppressed the effects of HBO treatment (^※^*p* < 0.05, *n* = 4)

To investigate the roles of miR-573 in cell proliferation of degenerative NP cells after HBO treatment, the MTT assay was employed. As shown in Fig. 5, overexpression of miR-573 by HBO treatment markedly increased NP cells proliferation (HBO/control: 1.63 ± 0.18-fold, **p < 0.01, *n* = 4), whereas transfection with anti-miR-573 partly suppressed the effects of HBO treatment (HBO + anti-miR573/control: 1.33 ± 0.12-fold, ^**※**^*p* < 0.05, *n* = 4).

### Overexpression of miR-573 by HBO treatment blocks the mitochondrial apoptotic pathway

**Fig. 6:**
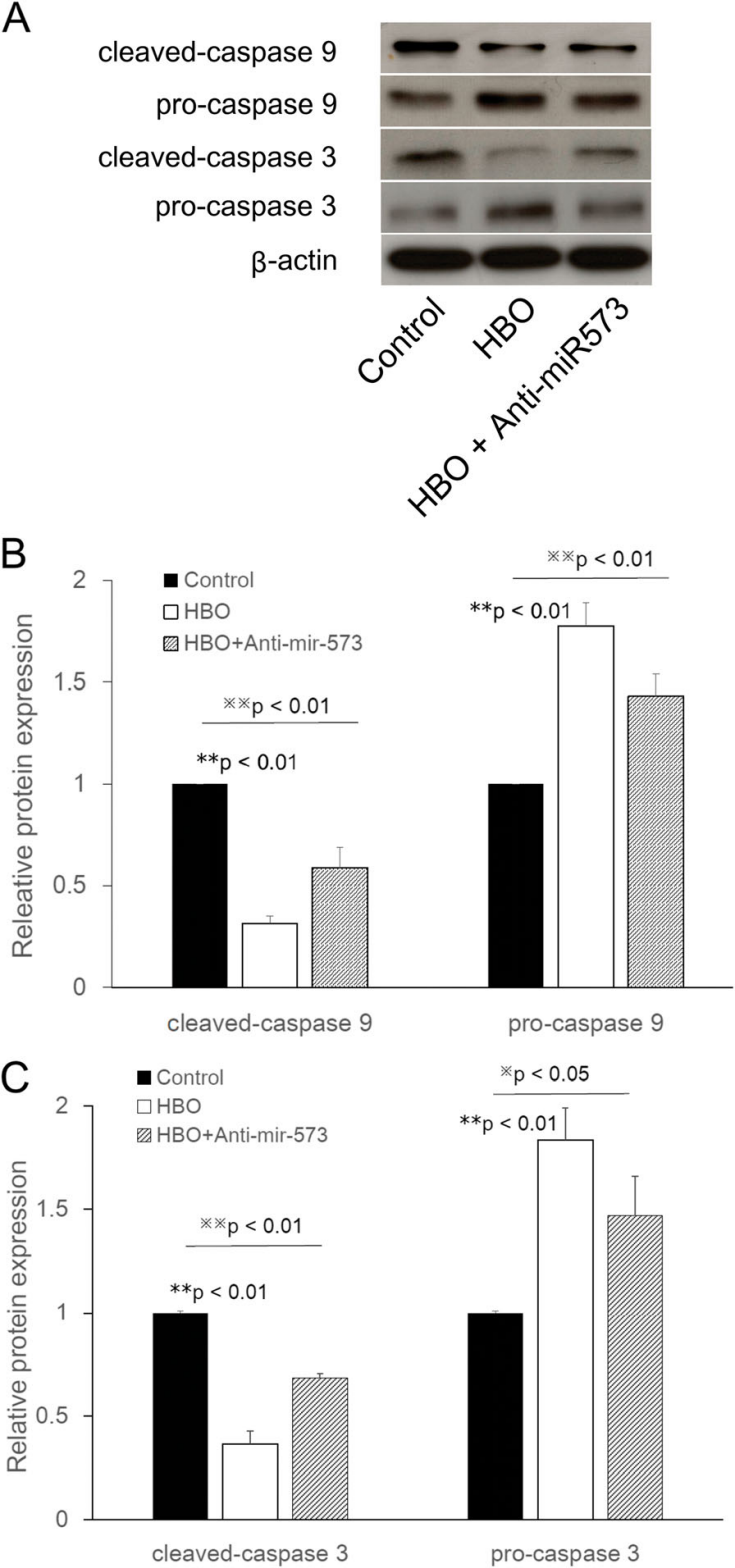
Overexpression of miR-573 by HBO treatment blocks the mitochondrial apoptotic pathway. HBO treatment significantly down-regulated the cleaved-caspase-9 (**a**, **b**, **p < 0.01, *n* = 4) and cleaved-caspase-3 (**a**, **c**, **p < 0.01, *n* = 4) expression, whereas transfection with anti-miR-573 partly suppressed the effects of HBO treatment (**b**, **c**, ^※※^*p* < 0.01, *n* = 4). At the same time, HBO treatment significantly reduced the activation (cleavage) of pro-caspase 9 (**a**, **b**, **p < 0.01, *n* = 4) and pro-caspase 3 proteins (**a**, **c**, **p < 0.01, *n* = 4), whereas transfection with anti-miR-573 partly suppressed the effects of HBO (**b**, **p < 0.01; **c**, ^※^*p* < 0.05, *n* = 4) in NP cells

To investigate whether miR-573 may regulate the mitochondrial apoptotic pathway by suppressing apoptotic-associated protein expression in NP cells following HBO treatment, western blotting was used to detect the cleaved-caspase 9, pro-caspase 9, cleaved-caspase 3, and pro-caspase 3 expression levels in NPCs. It was observed that HBO treatment significantly downregulated the cleaved-caspase 9 and cleaved-caspase-3 expression, whereas transfection with anti-miR-573 partly suppressed the effects of HBO treatment (Fig. 6a, b for cleaved-caspase 9: HBO/control: 0.32 ± 0.04-fold, **p < 0.01, *n* = 4; HBO + anti-miR-573/control: 0.59 ± 0.10-fold, ^**※※**^*p* < 0.01, *n* = 4; Fig. 6a, c for cleaved-caspase 3: HBO/control: 0.37 ± 0.06-fold, **p < 0.01, *n* = 4; HBO + anti-miR-573/control: 0.68 ± 0.02-fold, **p < 0.01, *n* = 4). At the same time, HBO treatment significantly reduced the activation (cleavage) of pro-caspase 9, whereas transfection with anti-miR-573 partly suppressed the effects of HBO treatment (Fig. 6a, b for pro-caspase 9: HBO/control: 1.78 ± 0.12-fold, **p < 0.01, *n* = 4; HBO + anti-miR-573/control: 1.43 ± 0.11-fold, ^**※※**^*p* < 0.01, *n* = 4 ) and pro-caspase 3 proteins (Fig. 6a, c for pro-caspase 3: HBO/control: 1.84 ± 0.15-fold, **p < 0.01, *n* = 4; HBO + anti-miR-573/control: 1.47 ± 0.18-fold, ^**※**^*p* < 0.05, *n* = 4) in NP cells.

## Discussion

Discectomy describes a class of operations used to remove part of an IVD or the entire IVD. Lumbar disc herniation (LDH) causes symptoms such as intermittent low back pain, sciatica, or patients may have more serious neurological symptoms. Lumbar microdiscectomies are always performed posteriorly (from the back) in LDH treatment, and often flavectomy and laminotomy are performed during this procedure [18, 19]. Degenerated disc hernia (extruded or migrated disc fragments) compressing the nerve roots and/or the spinal cord were harvested, and the amount of disc degeneration was graded according to Pfirrmann classification using T2-weighted MRI [20, 21].

Total discectomies, which remove the entire intervertebral disc, are usually done in concert with another procedure, such as artificial disc replacement or fusion. Total discectomies are done anteriorly (from the front). Recently, long-term data regarding total disc replacement (TDR ) surgery has become available, with some studies showing superior outcomes to fusion surgery [22]. In the present study, abnormal disk tissue was harvested from the degenerated lumbar IVD of 28 patients who receive total discectomy and posterior lumbar interbody fusion and the amount of disc degeneration was also graded according to Pfirrmann classification using T2 weighted MRI [20, 21]. Because cultures were prepared from tissues belonging to different patients in different age groups, the accuracy of the data may not be in doubt.

During degeneration, the oxygen concentration in the IVD becomes even lower than healthy IVD [13] and the expression level of miR-573 was downregulated notably in IDD tissues [15]. In addition, it has also been reported that miR-573 expression by breast cancer cells is downregulated in the presence of hypoxia [23]. MicroRNA microarray analysis showed a global regulation in mature miRNA levels in cells exposed to hypoxia [24] or hyperoxia [25]. There is some evidence that hypoxic disruption of miRNA biogenesis results in a global downregulation of miRNAs in hypoxia [24]. Our data indicated that miR-573 is one of the identified miRNAs upregulated in human degenerated NPCs following HBO treatment by microarray (Fig. 1a, b) and confirmed this expression via real-time PCR (Fig. 1c). HBO treatment increases the O_2_ levels to improve hypoxia conditions thus may regulate the expression of miRNAs.

Bioinformatics analysis showed that Bax [15], apolipoprotein M (apoM) [26], and E2F transcription factor 3 (E2F3) [27] was a potential target of hsa-miR-573 in diverse cells. Bax was the first identified pro-apoptotic member in the protein family of the B cell lymphoma-2 (Bcl-2) [28], and Bcl-2 is also a key anti-apoptotic protein [29]. Epigenetic knockdown of miR-143 regulated cell apoptosis in IDD by targeting Bcl-2 [30]. Low expression of miR-125a was found in IDD by targeting pro-apoptotic Bcl-2 antagonist killer 1 [31]. Furthermore, the expression of miR-573 was decreased whereas that of Bax was increased oppositely in human IDD tissues [15]. HBO treatment upregulates the ratio of Bcl-2 to Bax expression and reduced the apoptosis of ischemic tissue [32, 33] or degenerated NP cells [34]. In this study, we further found the epigenetic regulations of Bax mRNA expression. The 3′ UTR of Bax containing the miR-573 binding site (Fig. 2). HBO treatment decreased luciferase activity in the WT 3′ UTR of Bax compared with that of mutant forms (Fig. 3). Taken together, our present study suggested that Bax was a direct target of miR-573. Moreover, overexpression of miR-573 decreased the mRNA and protein levels of Bax (Fig. 4), which indicated a potential regulatory relationship between miR-573 and Bax following HBO treatment. HBO exerted its biological functions on NP cells via modulation of miR-573/Bax axis.

Increasing numbers of studies have demonstrated that miRNAs play vital roles in diverse pathological and biological processes by impacting on cell proliferation and apoptosis [28]. MiR-98 increased cell proliferation in NP cells [30]. Overexpression of miR-573 by mimic transfection increased cell viability in degenerated NP cells [15]. However, miR-573 upregulation inhibited melanoma cell proliferation also has been reported [35]. HBO treatment may promote [36–38] or inhibit [39] cell proliferation in diverse cell types. In consistent with previous studies, overexpression of miR-573 by HBO treatment markedly increased NP cells proliferation, whereas transfection with anti-miR-573 partly suppressed the effects of HBO treatment in the present study (Fig. 5). MiR-573 overexpression increased cell proliferation and suppressed cell apoptosis in degenerative NP cells following HBO treatment.

Mitochondria serve an important role in the apoptotic process by releasing apoptogenic molecules, including cytochrome *c* [40, 41]. Bax has been identified to have a pro-apoptotic effect, which may open the permeability transition pore on the mitochondrial membrane to trigger the release of cytochrome *c* from mitochondria into the cytoplasm [42]. Cytochrome *c* normally functions as a part of the respiratory chain, but when released into the cytosol, it becomes a critical component of the apoptosis execution machinery, where it activates caspases and causes apoptotic cell death. Cytochrome *c* may trigger the caspase-9-molulated cascade amplification reaction in the mitochondrial apoptotic pathway, which in turn processes pro-caspase 3 to generate active caspase 3 [43]. The present results suggested that upregulation of miR-573 decreased the Bax (Fig. 4), cleaved-caspase 9, and cleaved-caspase 3 expression levels and reduced the activation (cleavage) of pro-caspase 9 and pro-caspase 3 expression levels, whereas transfection with anti-miR-573 partly suppressed the effects of HBO treatment (Fig. 6). Collectively, these results suggested that miR-573 may exert its therapeutic effects on degenerated NP cells following HBO treatment by blocking the mitochondrial apoptotic pathway. However, there are some limitations in this study. Because other miRNAs may also be relevant for the regulation of apoptosis in NP cells following HBO treatment, transfection with anti-miR-573 only partly suppressed the effects of HBO treatment. In the future, further systematic and in-depth studies investigating the apoptosis in NP cells following HBO treatment will be conducted.

In conclusion, the results of this study indicate that HBO treatment of degenerated NP cells exerts a protective effect by mitigating apoptosis and its activation. Mir-573 regulates cell proliferation and apoptosis by targeting Bax in human degenerative NP cells following HBO treatment. Recently, HBO therapy in clinical patients with full endoscopic lumbar diskectomy (FELD) has been reported [44]. The clinical applications of HBO in degenerative IVD diseases are needed to be further investigated.

## Data Availability

We declare that the materials described in the manuscript will be freely available to all scientists for non-commercial purposes.

## Abbreviations

IDD: Intervertebral disc degeneration
NP cells: Nucleus pulposus cells
miRNAs: MicroRNAs
3′ UTR: 3′ Untranslated regions
mRNAs: Messenger RNAs
HBO: Hyperbaric oxygen
MTT: 3-(4,5-dimethyl-2-thiazolyl)-2,5-diphenyl-2H tetrazolium bromide
WT: Wild type
Mut: Mutant variant
apoM: Apolipoprotein M
E2F3: E2F transcription factor 3
